# Assessment of Imaging Modalities Against Liver Biopsy in Nonalcoholic Fatty Liver Disease: The Amsterdam NAFLD‐NASH Cohort

**DOI:** 10.1002/jmri.27703

**Published:** 2021-05-15

**Authors:** Marian A. Troelstra, Julia J. Witjes, Anne‐Marieke van Dijk, Anne L. Mak, Oliver Gurney‐Champion, Jurgen H. Runge, Diona Zwirs, Daniela Stols‐Gonçalves, Aelko H. Zwinderman, Marije ten Wolde, Houshang Monajemi, Sandjai Ramsoekh, Ralph Sinkus, Otto M. van Delden, Ulrich H. Beuers, Joanne Verheij, Max Nieuwdorp, Aart J. Nederveen, Adriaan G. Holleboom

**Affiliations:** ^1^ Department of Radiology and Nuclear Medicine Amsterdam University Medical Centres Amsterdam The Netherlands; ^2^ Department of Internal and Vascular Medicine Amsterdam University Medical Centres Amsterdam The Netherlands; ^3^ Department of Clinical Epidemiology, Biostatistics and Bioinformatics Amsterdam University Medical Centres Amsterdam The Netherlands; ^4^ Department of Internal Medicine Flevoziekenhuis Almere The Netherlands; ^5^ Department of Internal Medicine Rijnstate Ziekenhuis Arnhem The Netherlands; ^6^ Department of Gastroenterology and Hepatology Amsterdam University Medical Centres Amsterdam The Netherlands; ^7^ Inserm U1148, LVTS University Paris Diderot, University Paris 13 Paris France; ^8^ School of Biomedical Engineering and Imaging Sciences King's College London London UK; ^9^ Department of Pathology Amsterdam University Medical Centres Amsterdam The Netherlands

**Keywords:** nonalcoholic fatty liver disease, nonalcoholic steatohepatitis, magnetic resonance imaging, multiparametric, liver biopsy

## Abstract

**Background:**

Noninvasive diagnostic methods are urgently required in disease stratification and monitoring in nonalcoholic fatty liver disease (NAFLD). Multiparametric magnetic resonance imaging (MRI) is a promising technique to assess hepatic steatosis, inflammation, and fibrosis, potentially enabling noninvasive identification of individuals with active and advanced stages of NAFLD.

**Purpose:**

To examine the diagnostic performance of multiparametric MRI for the assessment of disease severity along the NAFLD disease spectrum with comparison to histological scores.

**Study Type:**

Prospective, cohort.

**Population:**

Thirty‐seven patients with NAFLD.

**Field Strength/Sequence:**

Multiparametric MRI at 3.0 T consisted of magnetic resonance (MR) spectroscopy (MRS) with multi‐echo stimulated‐echo acquisition mode, magnitude‐based and three‐point Dixon using a two‐dimensional multi‐echo gradient echo, MR elastography (MRE) using a generalized multishot gradient‐recalled echo sequence and intravoxel incoherent motion (IVIM) using a multislice diffusion weighted single‐shot echo‐planar sequence.

**Assessment:**

Histological steatosis grades were compared to proton density fat fraction measured by MRS (PDFF_MRS_), magnitude‐based MRI (PDFF_MRI‐M_), and three‐point Dixon (PDFF_Dixon_), as well as FibroScan® controlled attenuation parameter (CAP). Fibrosis and disease activity were compared to IVIM and MRE. FibroScan® liver stiffness measurements were compared to fibrosis levels. Diagnostic performance of all imaging parameters was determined for distinction between simple steatosis and nonalcoholic steatohepatitis (NASH).

**Statistical Tests:**

Spearman's rank test, Kruskal–Wallis test, Dunn's post‐hoc test with Holm‐Bonferroni *P*‐value adjustment, receiver operating characteristic curve analysis. A *P*‐value <0.05 was considered statistically significant.

**Results:**

Histological steatosis grade correlated significantly with PDFF_MRS_ (*r*_*s*_ = 0.66, *P* < 0.001), PDFF_MRI‐M_ (*r*_*s*_ = 0.68, *P* < 0.001), and PDFF_Dixon_ (*r*_*s*_ = 0.67, *P* < 0.001), whereas no correlation was found with CAP. MRE and IVIM diffusion and perfusion significantly correlated with disease activity (*r*_*s*_ = 0.55, *P* < 0.001, *r*_*s*_ = −0.40, *P* = 0.016, *r*_*s*_ = −0.37, *P* = 0.027, respectively) and fibrosis (*r*_*s*_ = 0.55, *P* < 0.001, *r*_*s*_ = −0.46, *P* = 0.0051; *r*_*s*_ = −0.53, *P* < 0.001, respectively). MRE and IVIM diffusion had the highest area‐under‐the‐curve for distinction between simple steatosis and NASH (0.79 and 0.73, respectively).

**Data Conclusion:**

Multiparametric MRI is a promising method for noninvasive, accurate, and sensitive distinction between simple hepatic steatosis and NASH, as well as for the assessment of steatosis and fibrosis severity.

**Level of Evidence:**

2

**Technical Efficacy:**

2

Along with the global increase in obesity and type 2 diabetes mellitus (T2DM), the prevalence of nonalcoholic fatty liver disease (NAFLD) is rising; in fact, NAFLD is currently the most common cause of liver dysfunction worldwide.^1^ It represents a spectrum of liver disease ranging from simple hepatic steatosis, through nonalcoholic steatohepatitis (NASH) to advanced fibrosis and cirrhosis. Advanced stages may ultimately be complicated by hepatocellular carcinoma.^2^

The overall global prevalence of NAFLD is estimated to be around 25% in the general population, while 60% of individuals with T2DM have NAFLD. Approximately 25% of individuals with simple steatosis are found to progress to NASH.^1^ The transition from simple steatosis to NASH and especially the subsequent development of fibrosis is associated with an increased risk of cardiovascular disease and mortality from liver‐related disease.^3^, ^4^ Given the potential clinical consequences, it is of crucial importance to differentiate simple steatosis from the active form of the disease (NASH) in order to identify and closely follow up progressors along the NAFLD disease spectrum.^5^

Currently, liver biopsy remains the diagnostic reference standard for NAFLD, as it is the most reliable test to distinguish simple steatosis from NASH and to assess the degree of inflammation and fibrosis.^6^ However, a liver biopsy has apparent disadvantages: It is invasive with a small risk of complications (most notably bleeding) and potentially painful and labor‐intensive, thus hampering its application in large‐scale studies. In addition, it is prone to sampling error as biopsies consist of only a focal assessment of the liver.^7^, ^8^ These drawbacks render liver biopsy a suboptimal diagnostic method for screening and monitoring of NAFLD in clinical practice.

Therefore, noninvasive methods that accurately assess hepatic fat content and even hepatic inflammation and fibrosis are in development for risk stratification of disease severity in NAFLD. Recently, Eddowes et al demonstrated in a biopsy‐controlled study that controlled attenuation parameter (CAP) and liver stiffness measurement (LSM) determined with vibration‐controlled transient elastography on the FibroScan® device accurately assesses steatosis and fibrosis, respectively, in individuals with NAFLD.^9^ However, this method is unable to capture the active form of the disease, that is, steatohepatitis.^10^ Therefore, magnetic resonance imaging (MRI) has been proposed as a tool to assess the full spectrum of NAFLD.^11^ While many studies have compared a single MRI sequence to a histopathological outcome, few studies have assessed the full NAFLD disease spectrum.

Multiparametric MRI is a promising technique to assess hepatic steatosis as well as inflammation and fibrosis, potentially enabling noninvasive identification of individuals with active and advanced stages of NAFLD. Thus the aim of our current proof‐of‐principle study is to assess the use of multiparametric MRI and determine the best‐performing imaging parameters for identifying the full NAFLD disease spectrum in a subgroup of the Amsterdam NAFLD‐NASH cohort.

## Materials and Methods

### Design

The study protocol was reviewed and approved by the institutional review board of the *Amsterdam University Medical Centres, location Academic Medical Centre (AMC)* and was registered in the Dutch Trial Register (registration number NTR7191). All participants in this study provided written informed consent. The Amsterdam NAFLD‐NASH cohort (ANCHOR) study is an observational prospective study that aims to identify and validate noninvasive diagnostic methods, both imaging and molecular markers, for the assessment of the entire NAFLD disease spectrum. To this end, participants undergo multiparametric MRI of the liver, hepatic FibroScan®, ultrasound guided liver biopsy, and urine, feces and blood sampling both at baseline and during follow‐up after 5 years. The study was begun at the end of 2018 and is still ongoing. It is conducted at *the AMC*, in compliance with the principles in the declaration of Helsinki and according to Good Clinical Practice guidelines.

### Participants

The first 37 individuals from the ANCHOR study were included in this proof‐of‐principle analysis. Individuals from *Amsterdam UMC* outpatient clinics with hepatic steatosis on abdominal ultrasound performed for other clinical reasons were included. The other main inclusion criteria were age above 18 years, levels above the upper limit of normal for either aspartate aminotransferase and/or alanine aminotransferase and a body mass index (BMI) above 25 kg/m^2^. Exclusion criteria were contraindications for undergoing MRI, excessive alcohol use (women >14 units/week, men >21 units/week), known bleeding disorders, the use of anticoagulant therapy and platelet aggregation inhibitors, diagnosis of decompensated liver cirrhosis and/or hepatocellular carcinoma, the use of drugs with a potential role in aggravation of pre‐existing NAFLD, and other known causes of liver steatosis other than NAFLD (auto‐immune hepatitis, hemochromatosis, hepatitis B and/or C, Wilson's disease, alpha‐1‐antitrypsin deficiency).

### MRI Acquisition

All individuals underwent multiparametric MRI of the liver using a clinical 3.0 T MRI unit (Ingenia; Philips, Best, the Netherlands) using a 16‐channel phased‐array anterior coil and a 10‐channel phase‐arrayed posterior coil. All data were acquired in a single approximately 45‐minute session. Participants were required to fast for a minimum of 4 hours before scanning. Images were analyzed by a single observer (*M.T*.) with 3 years of experience in hepatic MRI, who was blinded to the histopathology results.

To quantify the grade of hepatic steatosis, magnetic resonance spectroscopy (MRS), magnitude‐based MRI (MRI‐M) proton density fat fraction (PDFF), and three‐point Dixon were performed. MRS data acquisition was performed using a multi‐echo stimulated‐echo acquisition mode, according to our previously described protocol.^12^ MRI‐M and three‐point Dixon consisted of a two‐dimensional multi‐echo gradient echo sequence with, respectively, six and three echo times (TE). Three‐point Dixon sequence acquired both magnitude and phase data, while MRI‐M acquired only magnitude data.

Disease activity and fibrosis were quantified using intravoxel incoherent motion (IVIM) imaging and magnetic resonance elastography (MRE). The IVIM sequence consisted of a free‐breathing multislice diffusion weighted single‐shot echo‐planar imaging sequence with 18 unique *b*‐values. MRE was performed using a gravitational transducer at 50 Hz and a generalized multishot gradient‐recalled echo (Ristretto MRE).^13^

All imaging sequences were assessed for performance in distinguishing simple steatosis from NASH.

MRI acquisition parameters and sequence details for all included sequences are listed in Table 1 and in Appendix S1 in the Supplemental Material, respectively.

**TABLE 1:** Overview of Magnetic Resonance Imaging (MRI) Acquisition for Each Imaging Sequence Included

Parameter	MRS	MRI‐M	Three‐Point Dixon	MRE	IVIM
MRS voxel size (mm^3^)	20 × 20 × 20	–	–	–	–
Field of view (mm^2^)	–	448 × 320	420 × 300	448 × 448	450 × 295
Resolution (mm^2^)	–	4.0 × 4.0	2.4 × 2.4	4.0 × 4.0	3.0 × 3.0
Slice thickness (mm)	–	5	10	4	6
Slice gap (mm)	–	0	11.4	0	1
Slices	–	36	5	9	27
Parallel imaging SENSE factor	–	2	–	3	1.3
Partial averaging factor	–	–	–	–	0.6
Repetition time (msec)	3500	150	50	75	7000
Echo time (msec)	10, 15, 20, 25, 30	1.15, 2.33, 3.51, 4.69, 5.87, 7.05	3.1, 3.88, 4.66	6.91	45.5
Flip angle (°)	–	10	5	20	90
Bandwidth	2000 Hz	1666 Hz	436 Hz	2146 Hz	20.8 Hz/pixel
Acquisition duration	21 second breath‐hold	18 second breath‐hold	19 second breath‐hold	4 × 15 second breath‐hold	8.01 minute free‐breathing
Other	1024 data points; pencil beam volume B0 shimming	–	–	Four wave‐phase offsets; MEG frequency 165 Hz; Hadamard encoding; Ristretto sequence; gravitational transducer 50 Hz	*b*‐values: 0, 1, 2, 5, 10, 20, 30, 40, 50, 75, 100, 150, 200, 300, 400, 500, 600, 700 seconds/mm^2^; SPAIR fat suppression

MRS = magnetic resonance spectroscopy; MRI‐M = magnitude‐based MRI; MRE = magnetic resonance elastography; IVIM = intravoxel incoherent motion; MEG = motion encoding gradient.

### MRI Analysis

#### MRS PDFF (PDFF_MRS_)

MRS data analysis was performed using our previously described protocol.^12^ Spectral data were fitted using the AMARES algorithm^14^ in jMRUI version 4.0 (http://www.jmrui.eu/).^15^ PDFF_MRS_ values for each subject were calculated using the T2‐corrected water and combined fat peak amplitudes, after correcting for the amplitudes of fat peaks overlapping the water peak.^16^

#### MRI‐M PDFF (PDFF_MRI‐M_)

Three regions of interest (ROI) in the magnitude images of the right hepatic lobe were selected in three different slices, avoiding large vessels, bile ducts, and liver edges. Mean signal intensity per TE was determined and the PDFF calculated in Matlab R2018a (Mathworks, Natick, MA, USA) using a multi‐echo and multifrequency fat signal model to correct for T2* effects.^17^ The mean PDFF_MRI‐M_ of all three ROIs was used to establish an average fat percentage of the liver.

#### THREE‐POINT DIXON PDFF (PDFF_DIXON_ )

Magnitude images were used to draw ROIs of the liver in all slices, avoiding major blood vessels, bile ducts, and liver edges. Image analysis was performed in Matlab, using a toolbox with multipoint fat‐water separation using a hierarchical field map estimation^18^ from the ISMRM Fat‐Water Toolbox 2012 (https://github.com/maxdiefenbach/MRI_field_contributions/tree/master/fwtoolbox_v1_code). Both phase and magnitude images were used for the reconstruction. The ROI was overlaid on the reconstructed fat image to determine an average fat PDFF_Dixon_ of the entire liver in all five slices.

#### MAGNETIC RESONANCE ELASTOGRAPHY

Images were analyzed using ROOT software^19^ as described previously by Green et al.^20^ Phase images were locally unwrapped and filtered using a Gaussian filter (width σ 0.5). Viscoelastic parameter maps were reconstructed using a finite element‐based inversion algorithm.^21^ Mean stiffness based on the shear modulus (*G'*)) and loss modulus (*G"*) was determined from an ROI drawn in the middle of three slices, proximal to the transducer, avoiding large vessels and liver edges.^13^

#### IVIM IMAGING

ROIs were drawn in all slices containing liver tissue in the reconstructed combined *b*‐value images, avoiding large vessels, bile ducts, and liver edges. Slices or areas with artifacts were excluded from the mask. Diffusion (*D*), pseudo‐diffusion (D*), and perfusion fraction (*F*) parameter maps of the masked regions were compiled in MATLAB using a Bayesian‐probability based fit.^22^ Mean values across the masked liver area were reported for *D*, D*, and *F*.

### FibroScan® CAP and LSM

FibroScan® examinations were performed by a physician (J.W., A.v.D. or A.M.) who performed over 50 examinations and was blinded to the participants' histological evaluation. The device used was a FibroScan® 530 Compact (Echosens, France) equipped with both the M‐ and XL‐probe. The type of probe used depended on the real‐time assessment of the skin‐to‐liver capsule distance for each participant. The measurement was obtained in fasting condition of at least 2 hours, with the participant in a supine position with their right arm fully abducted. Measurements were performed in the right liver lobe through a midaxillary intercostal space. FibroScan® was performed either at the moment of screening, within a month of the MRI and liver biopsy, or on the day of MRI. By using transducer‐induced vibrations, the FibroScan® captures simultaneous recordings of CAP to quantify hepatic steatosis, and transient elastography to obtain LSM as an indicator of the presence of liver fibrosis.^23^

### Liver Biopsy

Percutaneous ultrasound‐guided liver biopsies were performed by either an interventional radiologist or a hepatologist according to local standard procedure. All histologic specimens were scored by a liver pathologist (J.V., 15 years of experience), who was blinded to all other data. Biopsy samples were stained using a hematoxylin and eosin stain and a Sirius Red stain. The histological parameters were defined with the use of the steatosis, activity, and fibrosis (SAF) score,^24^ classifying non‐NAFLD, simple steatosis, or NASH. The SAF‐score graded the degree of steatosis by the percentage of hepatocytes containing large and medium‐sized intracytoplasmic lipid droplets, on a scale of 0–3 (0: <5%; 1: 5–33%; 2: 34–66%; 3: >67%); hepatocellular ballooning was graded from 0 to 2 (0: normal hepatocytes; 1: clusters of hepatocytes with rounded shape and pale cytoplasm, but normal size; 2: as for grade 1, but with at least one enlarged/ballooned hepatocyte); and lobular inflammation was defined as a focus of two or more inflammatory cells within the lobule organized either as microgranulomas or located within the sinusoids (grade 0: none; 1: ≤2 foci per lobule; 2: >2 foci per lobule). NAFLD was defined by the presence of steatosis in at least 5% of the hepatocytes. NASH was diagnosed in cases with steatosis where hepatocellular ballooning grade was ≥1 and where lobular inflammation grade ≥1 was present. Activity grade (A0–4) was the unweighted addition of hepatocyte ballooning and lobular inflammation. Fibrosis was scored according to the NASH Clinical Research Network (CRN)^25^ as follows: stage 0 (F0) no fibrosis; stage 1 (F1): 1a or 1b perisinusoidal zone 3 or 1c periportal fibrosis; stage 2 (F2): perisinusoidal and periportal fibrosis without bridging; stage 3 (F3): bridging fibrosis; and stage 4 (F4): cirrhosis.

### Statistical Analysis

Statistical analysis was performed using R version 3.6.3.^26^ For baseline differences between two groups, unpaired Student's *t* test or the Mann–Whitney *U* test was used depending on the distribution of the data. Normality was tested according to the Shapiro–Wilk's method. Differences in median or means between the simple steatosis and NASH groups were determined for all imaging parameters, expressed as mean ± standard deviation or median with interquartile range. Spearman's rank test was used for correlation analysis, as all parameters were nonparametric. A *P*‐value <0.05 was considered statistically significant. Comparison of medians for analysis of multiple groups was performed using the Kruskal–Wallis test, followed by Dunn's post‐hoc test with Holm‐Bonferroni *P*‐value adjustment when appropriate. Correlations and comparison of medians were assessed for steatosis grade compared to PDFF_MRS,MRI‐M,Dixon_ and FibroScan® CAP; activity grade compared to IVIM and MRE; and fibrosis grade compared to IVIM, MRE, and FibroScan® LSM. Receiver operating characteristic (ROC) curve analysis was used to determine the diagnostic performance of imaging parameters, reporting the area under the ROC (AUROC) and optimal cut‐off values with sensitivity and specificity. ROC analysis was used to assess the performance of PDFF_MRS,MRI‐M,Dixon_ and FibroScan® CAP for distinguishing between steatosis S1 and S2–3; MRE and IVIM for distinguishing between no/mild fibrosis (F0–F2) and advanced fibrosis (F3–F4). Finally, ROC analysis was reported for determining the performance of all imaging parameters for stratifying individuals in simple steatosis and NASH groups. In case of missing data, the analysis was performed for all available parameters.

## Results

### Inclusion

Between September 2018 and October 2020, a total of 37 individuals (23 men, 14 women) with hepatic steatosis on ultrasound were included. Mean age of the participants was 49.0 years (SD ± 13.2) and mean BMI was 33.2 kg/m^2^ (SD ± 3.8). No adverse events were recorded during the MRI or liver biopsy. Liver biopsy was performed within 1 week of the MRI, with the exception of one participant whose biopsy was performed 2 months after the MRI. MRE data were not available for two individuals due to technical issues with the MRE hardware, one IVIM scan was not included in the analysis due to a low signal‐to‐noise ratio, and data were missing for one PDFF_Dixon_. Clinical characteristics of the participants are shown in Table 2.

**TABLE 2:** Clinical Characteristics of 37 Individuals With Biopsy‐Proven Nonalcoholic Fatty Liver Disease

	Reference Range	Study Participants, *N* = 37
Age (years)		49.0 ± 13.2
Male gender (%)		62
BMI (kg/m^2^)		33.2 ± 3.8
Type 2 diabetes mellitus (%)		43
Glucose (mmol/L)	3.5–6.1	5.9 [5.4–8.2]
ASAT (IU/L)	0–40	41.5 [37.0–51.0]
ALAT (IU/L)	0–40	59 [46.5–79.0]
ALP (IU/L)	40–120	88.0 ± 29.0
g‐GT (IU/L)	6–50	62.0 [42.0–88.0]
Cholesterol (mmol/L)	3–5.5	5.2 ± 1.0
HDL‐C (mmol/L)	0.9–1.7	1.2 ± 0.3
LDL‐C (mmol/L)	1.6–4.8	3.0 ± 1.0
Triglycerides (mmol/L)	0.7–2.1	2.2 [1.3–2.5]
CRP (mg/mL)	0–5	2.9 [1.7–4.3]
Steatosis (%)		30.0 [20.0–50.0]
Fibrosis score		2.0 [1.5–2.0]

Data are expressed as mean ± standard deviation or median [interquartile range], depending on the distribution of the data.

BMI = body mass index; ALP = alkaline phosphatase; g‐GT = gamma glutamyl transferase; ALAT = alanine aminotransferase; ASAT = aspartate aminotransferase; HDL = high‐density lipoprotein; LDL = low‐density lipoprotein; CRP = C‐reactive protein.

### Liver Histology Results

All biopsies were included in the final analysis. The mean biopsy length was 18 mm (SD ± 5.5). Twenty participants fell in the S1 grade, 13 S2 and four S3. Disease activity according to the SAF score showed three individuals with no activity (A0), 12 A1, 16 A2, five A3, and one A4. According to the SAF‐score, NASH was present in 22 of the 37 participants. According to the NASH‐CRN criteria for fibrosis, two individuals exhibited no fibrosis (F0), seven F1, 20 F2, seven F3, and one cirrhosis (F4). Of the two individuals with missing MRE data, one had F2 and one F4. IVIM data were missing for an individual with F2.

Steatosis grade did not correlate with either disease activity grade (*r*_*s*_ = 0.26, *P* = 0.12) or fibrosis stage (*r*_*s*_ = −0.04, *P* = 0.81). Fibrosis grade did correlate significantly with disease activity score (*r*_*s*_ = 0.65, *P* < 0.001).

### Assessment of Steatosis

**FIGURE 1:**
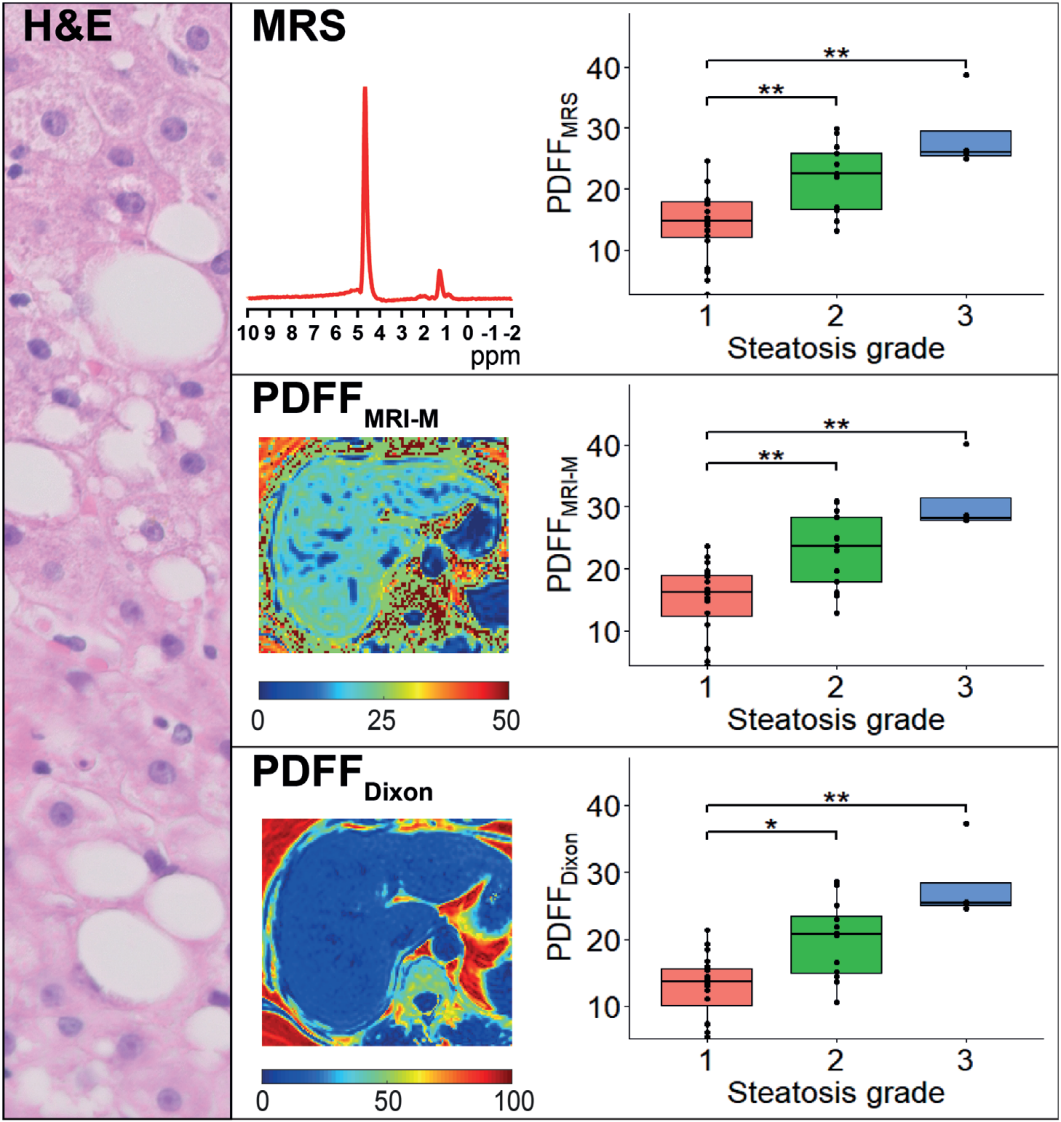
MRS and MRI based PDFF values vs. histological steatosis grade as seen in hematoxylin and eosin stain. Median PDFF values for grade 1, 2, and 3 were 14.8%, 22.5%, and 26.1%, respectively, for PDFF_MRS_; 16.2%, 23.7%, and 28.2%, respectively, for PDFF_MRI‐M_; and 13.6%, 20.7%, and 25.4%, respectively, for PDFF_Dixon_. All three parameters showed a significant difference in medians between grades 1–2 and grades 1–3.

Histological steatosis grade correlated significantly with PDFF_MRS_ (*r*_*s*_ = 0.66, *P* < 0.001), PDFF_MRI‐M_ (*r*_*s*_ = 0.68, *P* < 0.001), and PDFF_Dixon_ (*r*_*s*_ = 0.67, *P* < 0.001). Differences in hepatic fat fraction between grades of steatosis indicated a significant difference in the percentage of hepatic fat and steatosis grade (PDFF_MRS_: *χ* ^2^ = 15.75, *P* < 0.001, df = 2; PDFF_MRI‐M_: *χ* ^2^ = 16.54, *P* < 0.001, df = 2; PDFF_Dixon_: *χ* ^2^ = 15.99, *P* < 0.001, df = 2). All three methods showed a significant difference in medians between steatosis grade 1 and grade 2, and grade 1 and grade 3 **(**Fig. 1 **)**. In contrast, we found no correlation between histological steatosis grade and CAP measured by FibroScan® (*r*_*s*_ = 0.04, *P* = 0.82) **(**Fig. S1 in the Supplemental Material).

ROC analysis for differentiating S1 from S2–S3 showed similar values for all three MRI methods. PDFF_MRS_ had an AUROC of 0.86 (95% confidence interval (CI) 0.74–0.98) with a sensitivity of 70.6% and specificity 100% at a cut‐off value of 21.98%. PDFF_MRI‐M_ showed an AUROC of 0.87 (95% CI 0.75–0.99) with a sensitivity of 70.6% and specificity of 95.0% at a cut‐off of 22.93%. PDFF_Dixon_ had an AUROC of 0.86 (95% CI 0.74–0.99), with a sensitivity of 68.8% and specificity 95.0% at a cut‐off of 20.53%.

PDFF_MRS_, PDFF_MRI‐M_, and PDFF_Dixon_ strongly correlated among themselves (PDFF_MRS_ vs. PDFF_MRI‐M_: *r*(35) = 0.99, *P* < 0.001; PDFF_MRS_ vs. PDFF_Dixon_: *r*(34) = 0.98, *P* < 0.001; PDFF_MRI‐M_ vs. PDFF_Dixon_: *r*(34) = 0.99, *P* < 0.001).

### Assessment of Disease Activity: Lobular Inflammation and Hepatocyte Ballooning

#### MAGNETIC RESONANCE ELASTOGRAPHY

**FIGURE 2:**
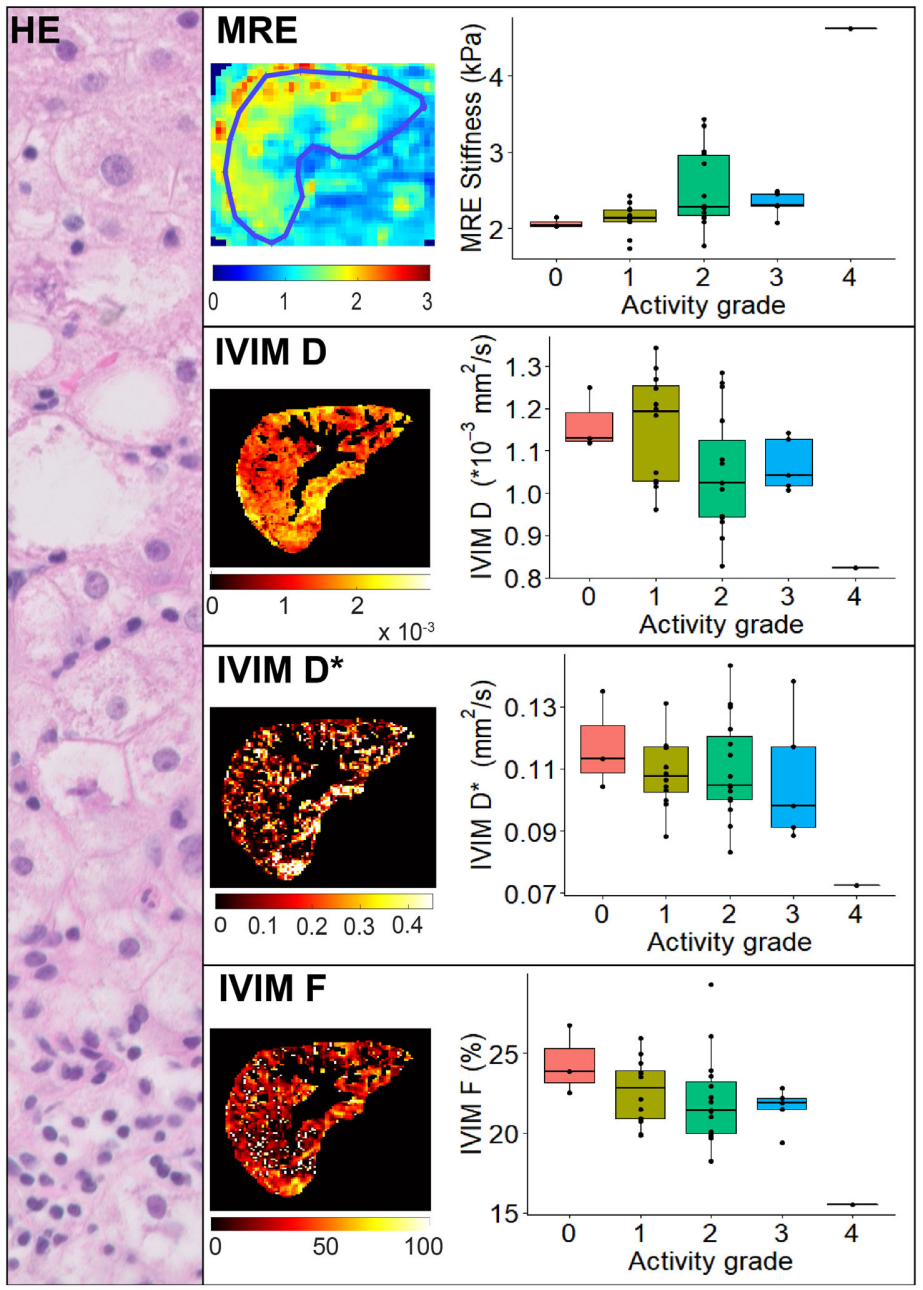
MRE and IVIM parameters vs. histological disease activity grade as seen in hematoxylin and eosin stain. MRE showed median stiffness values of: 2.04 kPa for grade 0, 2.14 kPa for grade 1, 2.29 kPa for grade 2, 2.31 kPa for grade 3, and a single value of 4.62 kPa for grade 4. Kruskal–Wallis tests indicated a significant difference in medians between activity grades; however, post‐hoc analysis did not provide a significant difference between individual grades. Median IVIM values for grade 0, 1, 2, 3, and 4 were 1.13, 1.19, 1.03, 1.04, and 0.83 × 10^−3^ mm^2^/second, respectively, for *D*; 0.11, 0.11, 0.10, 0.10, and 0.07 mm^2^/second, respectively, for D*; and 23.8, 22.8, 21.4, 21.9, and 15.5% for *F*. No IVIM parameters showed a significant difference in medians between activity grades.

*G'* showed a significantly positive correlation with activity grade (*r*_*s*_ = 0.55, *P* < 0.001), and comparison of stiffness results for individual activity grades showed a significant difference in medians between the groups (*χ* ^2^ = 11.02, df = 4, *P* = 0.026) **(**Fig. 2 **)**. *G"* did not show a correlation with activity grade (*r*_*s*_ = 0.28) or difference in medians between groups (*χ* ^2^ = 7.44, df = 4, *P* = 0.11).

#### INTRAVOXEL INCOHERENT MOTION

For both *D* (*r*_*s*_ = −0.40, *P* = 0.016) and *F* (*r*_*s*_ = −0.37, *P* = 0.027), we found a significant negative correlation with histological activity grade. D* had no significant correlation with activity grade. Kruskal–Wallis tests did not show a significant difference in medians between activity grades for any IVIM parameter (*D*: *χ* ^2^ = 8.00, df = 4, *P* = 0.11; D*: *χ* ^2^ = 4.05, df = 4, *P* = 0.40; *F*: *χ* ^2^ = 6.73, df = 4, *P* = 0.15) **(**Fig. 2 **)**.

#### FIBROSCAN® LSM

Liver stiffness measured by FibroScan® showed a significant correlation with histological activity grade (*r*_*s*_ = 0.43, *P* = 0.0077). However, comparison of medians did not show significant differences between activity grades (*χ* ^2^ = 7.58, df = 4, *P* = 0.11) **(**Fig. S2 in the Supplemental Material**)**.

Assessment of the individual components of the activity score revealed that MRE (*r*_*s*_ = 0.55, *P* < 0.001), IVIM *D* (*r*_*s*_ = −0.37, *P* = 0.025), IVIM *F* (*r*_*s*_ = −0.35, *P* = 0.037), and FibroScan® LSM (*r*_*s*_ = 0.45, *P* = 0.0087) correlated significantly with ballooning. In contrast, none of the available imaging modalities correlated with histopathological inflammation grade. Comparison of medians revealed a significant difference between ballooning grades for MRE (*χ* ^2^ = 10.46, df = 2) and FibroScan® LSM (*χ* ^2^ = 7.69, df = 2, *P* = 0.021). Inflammation grade did not show significant differences in medians for any imaging modality.

### Assessment of Fibrosis

#### MAGNETIC RESONANCE ELASTOGRAPHY

**FIGURE 3:**
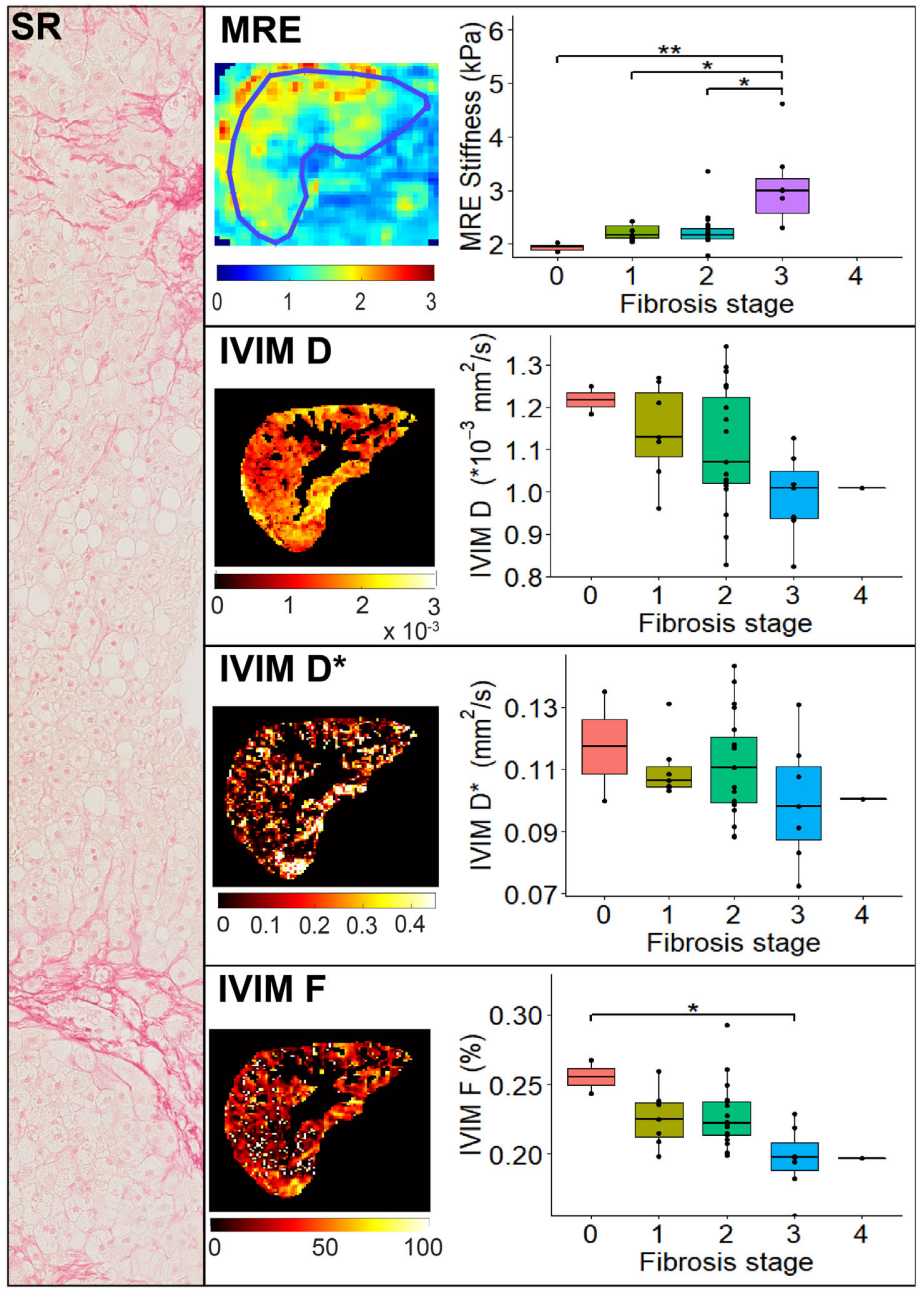
MRE and IVIM parameters vs. histological fibrosis grade as seen in Sirius Red stain. Median MRE stiffness values were: 1.93 kPa for grade 0, 2.16 kPa for grade 1, 2.17 kPa for grade 2, and 2.99 kPa for grade 3. There was a significant difference in medians between fibrosis grades 0–3, grades 1–3, and grades 2–3. Median IVIM values for grades 0, 1, 2, 3, and 4 were 1.22, 1.14, 1.10, 0.99, and 1.01 × 10^−3^ mm^2^/second, respectively, for *D*; 0.12, 0.11, 0.11, 0.10, and 0.10 mm^2^/second, respectively, for D*; and 25.5, 22.5, 22.2, 19.7, and 19.7%, respectively, for *F*. Only IVIM *F* showed a significant difference in medians between fibrosis grades 1–3.

We noted a significant positive association between MRE shear stiffness and the histological fibrosis score (*r*_*s*_ = 0.55, *P* < 0.001). Furthermore, we observed a significant difference between liver stiffness and fibrosis stage (*χ* ^2^ = 14.45, *P* = 0.0024, df = 3). Post‐hoc analysis showed a significant difference in liver stiffness between fibrosis stage F0–F3, F1–F3, and F2–F3 **(**Fig. 3 **)**. *G"* did not provide a significant correlation with fibrosis grade (*r*_*s*_ = 0.27, *P* = 0.11) or show significant differences between the various grades (*χ* ^2^ = 4.09, df = 3, *P* = 0.25).

For distinguishing individuals with no/mild (F0–F2) vs. advanced (F3, due to lack of F4 data in MRE cohort) fibrosis grade, the AUROC for MRE was 0.92 (95% CI 0.83–1), with a sensitivity of 100% and a specificity of 78.6% at an optimal cut‐off of 2.30 kPa. *G"* showed an AUROC of 0.74 (95% CI 0.48–1), with a sensitivity of 71.4% and specificity of 92.9% at an optimal cut‐off of 0.94 kPa.

#### INTRAVOXEL INCOHERENT MOTION

Significant correlations between IVIM parameters and fibrosis grade were found for two of the three parameters: *D* (*r*_*s*_ = −0.46, *P* = 0.0051) and *F* (*r*_*s*_ = −0.53, *P* < 0.001). D* did not show a significant correlation (*r*_*s*_ = −0.26, *P* = 0.13). Differences in medians for IVIM parameters between fibrosis stages only indicated a significant difference for *F* (*χ* ^2^ = 13.39, df = 4), and not for *D* (*χ* ^2^ = 7.78, *P* = 0.10, df = 4) or D* (*χ* ^2^ = 3.03, *P* = 0.55, df = 4). Post‐hoc analysis only showed a significant difference between the median *F* values of the F0 and F3 stages **(**Fig. 3 **)**.

Distinction between no/mild (F0–F2) and advanced (F3–F4) fibrosis for *D* resulted in an AUROC of 0.79 (95% CI 0.63–0.95), with a sensitivity of 75.0% and specificity of 78.6% at an optimal cut‐off value of 0.00102 mm^2^/second. D* had an AUROC of 0.70 (95% CI 0.47–0.93), with a sensitivity of 62.5% and specificity of 75.0% at a cut‐off of 0.10 mm^2^/second. *F* performed best out of the IVIM parameters for distinguishing between mild and advanced fibrosis with an AUROC of 0.88 (95% CI 0.72–1), sensitivity of 75.0%, and specificity of 100% at an optimal cut‐off at 19.85%.

#### FIBROSCAN® LSM

There was a significant correlation between LSM by FibroScan® and histological fibrosis grade (*r*_*s*_ = 0.47, *P* = 0.0034); however, differences in liver stiffness between stages of fibrosis indicated no significant difference between liver stiffness and fibrosis stage (*χ* ^2^ = 8.27, *P* = 0.082, df = 4) **(**Fig. S3 in the Supplemental Material**)**.

Distinction between no/mild (F0–F2) and advanced (F3–F4) fibrosis using FibroScan® LSM resulted in an AUROC of 0.77 (95% CI 0.58–0.96), with a sensitivity of 87.5% and specificity of 69.0% at an optimal cut‐off value of 9.9 kPa.

### Distinction Between Simple Steatosis and NASH

**FIGURE 4:**
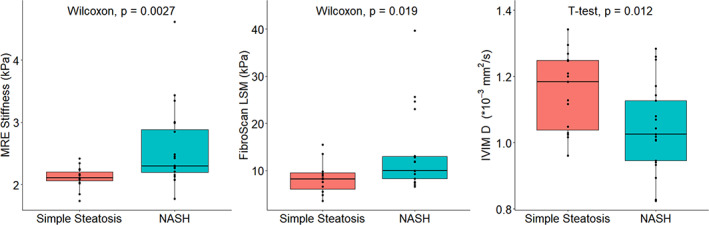
MRE *G*', FibroScan LSM and IVIM *D* parameters for simple steatosis vs. nonalcoholic steatohepatitis (NASH) individuals. Median MRE *G*' values were 2.11 kPa for simple steatosis and 2.30 kPa for NASH. Median FibroScan LSM values were 8.35 kPa for simple steatosis and 13.1 kPa for NASH. Median *D* values were 1.15 × 10^−3^ mm^2^/second for simple steatosis and 1.04 × 10^−3^ for NASH. All three parameters showed a significant difference in median/mean between the simple steatosis and NASH group, in contrast to other imaging parameters.

As reported under “Liver histology results,” NASH was histopathologically identified in 22 of the 37 participants using the SAF score. For MRE *G'* (*W* = 62, *P* = 0.0027), IVIM *D* (*t* = 2.66, df = 31.97, *P* = 0.012), and FibroScan® LSM (*W* = 89, *P* = 0.019) medians of the NASH group were all significantly different compared to the simple steatosis group **(**Fig. 4 **)**. IVIM *F*, D*, and steatosis imaging parameters did not correlate with the presence of NASH.

To determine the performance of each imaging modality for distinguishing individuals with NASH from those with simple steatosis, ROC analysis was performed. AUROC, sensitivity, and specificity values for each imaging modality are reported in Table 3. MRE *G*' showed the highest AUROC of 0.79, followed by FibroScan LSM and IVIM *D* (both 0.73). Of these, specificity was highest for both MRE and Fibroscan LSM at 86.7%, while IVIM *D* showed the highest sensitivity at 85.7%.

**TABLE 3:** Diagnostic Performance of Imaging Parameters in Distinguishing Between Simple Steatosis and Nonalcoholic Steatohepatitis

	AUROC	Cut‐Off Values	Sensitivity (%)	Specificity (%)
MRE *G*'	0.79	2.27 kPa	70	86.7
FibroScan® LSM	0.73	9.9 kPa	63.6	86.7
IVIM *D*	0.73	0.0012 mm ^2^ /second	85.7	53.3
MRE *G*"	0.69	0.88 kPa	45	100
IVIM *F*	0.68	22.90%	81	53.3
FibroScan® CAP	0.65	336 m/second	77.3	53.3
IVIM D*	0.58	0.10 mm ^2^ /second	47.6	80
PDFF_MRI‐M_	0.57	23.72%	40.9	86.7
PDFF_MRS_	0.56	21.19%	45.5	73.3
PDFF_Dixon_	0.52	21.84%	33.3	86.7

MRE = magnetic resonance elastography; LSM = liver stiffness measurement; IVIM *D* = intravoxel incoherent motion diffusion imaging; IVIM *F* = intravoxel incoherent motion perfusion fraction imaging; CAP = controlled attenuation parameter; IVIM D* = intravoxel incoherent motion pseudodiffusion imaging; PDFF_MRI‐M_ = magnitude‐based MRI proton density fat fraction; PDFF_MRS_ = magnetic resonance spectroscopy proton density fat fraction; PDFF_Dixon_ = three‐point DIXON proton density fat fraction.

## Discussion

The present proof‐of‐principle analysis shows that with the use of multiparametric MRI, NAFLD severity—particularly steatosis and fibrosis, and to a lesser extent disease activity—can be assessed. To evaluate the risk of progression toward advanced NASH and fibrosis and to determine treatment and monitoring plans, distinction between simple steatosis and NASH, and detection and staging of fibrosis are essential, yet challenging, components in the workup of an individual with NAFLD. This study presents the initial results from the Amsterdam NAFLD‐NASH cohort (ANCHOR) study, aimed at improving the noninvasive diagnosis and follow‐up of individuals with various grades and stages of NAFLD. Inclusion in the Amsterdam NAFLD cohort is still ongoing and future studies in this cohort will have to determine whether a combination of these MRI parameters can be used to predict histopathological outcomes. The current population captures the complete spectrum of NAFLD disease severity.

The noninvasive assessment of hepatic steatosis has been extensively studied for all of the imaging techniques used in this study: CAP, PDFF_MRS_, PDFF_MRI‐M_, and PDFF_Dixon._ ^9^, ^16^, ^17^, ^27^ Our findings are consistent with previous reports, showing strong correlations between steatosis grade and PDFF found with the MRI techniques. The strong correlation among the three MRI techniques would suggest that a single imaging technique for determining steatosis should be sufficient when performing multiparametric MRI.

Interestingly, FibroScan® CAP measurements did not correlate with histological steatosis grade, in contrast with previous studies.^9^, ^12^ At S1 steatosis grade, the CAP values showed a large spread in the, and medians of CAP did not differ between the three histological steatosis grades (S1–S3). This could potentially be due to our sample size and the relatively sizeable percentage of patients with S1 steatosis. In a recent cohort study, Eddowes et al found no difference in CAP between histological steatosis grades 2 and 3,^9^ indicating the small difference in CAP values between these groups, which may be observable only in large cohorts. Another explanation could be the nonlinear relationship of CAP values with the number of hepatocytes affected and grade of steatosis at biopsy,^12^ complicating the use of CAP as a tool to stage hepatic steatosis.

MRE *G'*, IVIM *D*, and IVIM *F* significantly correlated with the disease activity score, despite substantial overlap between values of the various levels. We also performed an analysis of the individual components of the activity score, comprised of inflammation and ballooning. MRE *G'*, IVIM *F*, IVIM *D*, and FibroScan® LSM showed significant correlations with ballooning, while only IVIM *D* correlated with inflammation. Previous studies have shown correlations between MRE *G'* measurements and inflammation and/or ballooning stages,^28^, ^29^ yet also with substantial overlap between severity grades. Loss modulus measured via MRE has shown promising results for evaluating inflammation levels,^30^, ^31^ but this correlation was not present in our cohort. Murphy et al showed a correlation between IVIM *F* and both inflammation and ballooning; however, those correlations did not uphold in a multivariable regression analysis.^32^ Poor correlations between imaging parameters and inflammation grade were likely caused by a relatively small spread in our cohort, with the majority exhibiting lobular inflammation grade 1 (*N* = 32). The imaging correlations with activity score were thus chiefly driven by the contrast in ballooning grade.

Staging individuals with NAFLD by fibrosis severity has clear prognostic consequences: progression into the fibrotic stages of NAFLD is strongly associated with liver‐related and overall mortality,^3^, ^4^ thus timely detection of NAFLD fibrosis is necessary. Here we show the capability of multiparametric MRI in differentiating between no/mild (F0–F2) and advanced (F3–F4) fibrosis.

Differences in MRE hardware, MRE scan acquisition techniques, vibrational frequencies, and postprocessing techniques make it difficult to directly compare stiffness values found with MRE between studies.^33^ No previous studies with MRE wave generation of 50 Hz in individuals with NAFLD are available; however, a larger retrospective study scanned at 60 Hz showed a similar diagnostic accuracy for distinguishing F0–F2 from F3–F4 (AUROC 0.954, sensitivity 0.85, specificity 0.929 at a cut‐off of 4.15 kPa).^34^

Diagnostic accuracy of LSM by FibroScan® for distinguishing no/mild fibrosis from advanced fibrosis was found to be inferior to MRE, as reported in previous studies.^35^ LSM accuracy in this study was slightly inferior to the diagnostic performance found in a recent study by Eddowes et al, reporting an AUROC of 0.80 (95% CI, 0.75–0.84).^9^

A recent review assessing correlations between IVIM and fibrosis grade showed substantial heterogeneity in found results.^36^ In the present study, IVIM *F* showed a strong correlation with fibrosis stage and showed good performance for distinguishing between no/mild and advanced fibrosis, yet issues with reproducibility shown in previous studies^37^ require further investigation.

Differentiating NASH from simple steatosis enables early detection of typically asymptomatic active disease. Currently available serum biomarkers for noninvasively differentiating simple steatosis from NASH such as alanine aminotransferase (ALT)‐levels and cytokeratin‐18 fragments are suboptimal, exhibiting poor reproducibility and low accuracy.^38^ The FAST score, however, a score that identifies patients with progressive NASH and has been validated in multiple large global cohorts, showed good performance with an AUROC ranging from 0.74 to 0.95.^10^ Previous MRI‐based studies have shown promising results for the distinction of simple steatosis from NASH using various techniques. T1‐based liver inflammation score showed an AUROC of 0.80; however, while the sensitivity was high at 91%, specificity proved to be limited at 52%.^39^ Ultrasmall superparamagnetic iron oxide particle (USPIO)–enhanced MRI found an AUROC of 0.87 in a group of 24 participants,^40^ yet concerns raised about the safety of USPIO administration^41^ make it less desirable as a diagnostic method. A previous MRE study showed an AUROC of up to 0.93 for distinguishing between NASH and simple steatosis using MRE shear stiffness, yet these findings suggest that this could be explained by the increase in liver stiffness caused by fibrosis.^28^ More recent work assessing MRE for identifying cases with NASH and fibrosis ≥2 suggests other results, with an AUROC of only 0.66.^42^ Hence, disease stratification in NAFLD is currently still reliant on liver biopsy, which comes with several disadvantages, as described in the introduction.

The current proof‐of‐concept study shows that the MRI parameters MRE *G'* and IVIM *D* have the potential to noninvasively differentiate simple steatosis from NASH. Of these methods, sensitivity was highest for IVIM *D* and specificity highest for MRE *G'*. Noninvasive methods with a high specificity such as MRE are particularly interesting as they have the potential to reduce the amount of liver biopsies in individuals with an early disease stage.

Development of models using a combination of imaging parameters, with or without ultrasonic parameters and plasma biomarkers to distinguish between simple steatosis, are of increasing interest. In recent work an AUROC of 0.87 was found combining MRE with PDFF for distinguishing between simple steatosis and NASH,^43^ while the combination of corrected T1 (cT1), aspartate aminotransferase (AST), and fasting glucose levels had an AUROC of 0.90 for identifying NASH with fibrosis ≥2.^44^ Finding the ideal combination of biomarkers will be the focus of further studies from the ANCHOR cohort.

### Limitations

We report the initial results from the ANCHOR cohort study with promising diagnostic performance of multiparametric MRI in NAFLD; however, while the current population captures the complete spectrum of NAFLD disease severity, not all groups were evenly represented, and the cohort size is modest. Multiple imaging modalities correlated with histopathology; however, the median values found with MRI often did not significantly differ between the histopathologically determined disease severity grades due to the limited number of subjects in some groups. Furthermore, combination of outcomes in a multivariate regression analysis will only be possible when the ANCHOR cohort has advanced in size in the upcoming years. This would be of additional value as single imaging parameters have often provided promising results, but either show overlapping values between the target groups or lack sensitivity and/or specificity for providing a definite diagnosis. While power analysis for distinction between simple steatosis and NASH shows that a sample size starting from 23 participants for the best performing imaging parameter should be sufficient, theory suggests that introduction of a logistical regression model would require a minimum of 100 participants,^45^ making this the minimum sample size for ensuing MRI studies from this cohort. Furthermore, MRE data for two individuals were unavailable, leaving no individuals with F4 fibrosis and limiting the assessment of its diagnostic accuracy in fibrosis severity. Finally, the use of a liver biopsy as a reference standard has the chance of sampling error and interobserver variability. This emphasizes the advantage of MRI, capturing the entire liver with quantitative measurements.

## Conclusion

Overall, our results indicate that multiparametric MRI is a promising noninvasive method for the accurate assessment of steatosis and fibrosis severity, as well as ballooning, with the potential to distinguish between simple hepatic steatosis and NASH.

## Supporting information


**Appendix S1**. Supporting Information

**Fig. S1** FibroScan® CAP values versus histological steatosis grade. Median values for grade 1, 2, and 3 were 324, 348, and 336 dB/m resp. There were no significant differences in medians between steatosis grades.

**Fig. S2** FibroScan® LSM versus histological activity grade. Median stiffness values were: 6.85 kPa for grade 0, 6.93 kPa for grade 1, 10.50 kPa for grade 2, and 11.1 kPa for grade 3. There was no significant difference in medians between activity grades.

**Fig. S3** FibroScan® LSM versus histological fibrosis grade. Median stiffness values were: 7.05 kPa for grade 0; 7.26 kPa for grade 1; 8.48 kPa for grade 2; 10.40 kPa for grade 3; and 23.00 kPa for grade 4. There were no significant differences in medians between fibrosis grades.
