# Supplementary material for: Assessment of Imaging Modalities Against Liver Biopsy in Nonalcoholic Fatty Liver Disease: The Amsterdam NAFLD‐NASH Cohort
Source: J Magn Reson Imaging. 2021 May 15;54(6):1937–49. doi: 10.1002/jmri.27703 (PMC9290703; doi:10.1002/jmri.27703)
Supplement: Supplementary file 1 — Appendix S1. Supporting Information [file JMRI-54-1937-s003.docx]

## MRI Acquisition

### *Magnetic resonance spectroscopy*

MRS data acquisition and analysis was performed using our previously described protocol^1^. Briefly, data were acquired using a multi-echo stimulated-echo acquisition mode (STEAM) in a single breath-hold of 21 seconds. A single voxel of 20×20×20mm^3^ was positioned in the right hepatic lobe, avoiding major blood vessels, bile ducts and liver margins. First order pencil beam volume B0 shimming was used. 1024 datapoints were acquired at a bandwidth of 2000Hz at five seperate echo times (TE) of 10, 15, 20, 25 and 30ms and a repetition time (TR) of 3500ms.

### *Magnitude-based MRI PDFF*

MRI-M for determination of PDFF was performed using a two-dimensional multi-echo gradient echo sequence. Six echo times were used, with an initial TE of 1.15ms, and ΔTE of 1.18ms. A flip angle of 10° and a repetition time of 150ms was used for all scans. The field-of-view (FOV) was kept the same throughout all individuals, set at 448×320mm^2^, 4.0×4.0mm^2^ acquisition resolution, 36 slices and 5mm slice thickness. Parallel imaging (SENSE) was used to accelerate data acquisition using a SENSE factor of 2, resulting in full liver coverage in a single 18-second breath-hold.

### *Three-point DIXON*

Three-point Dixon scans were performed using a two-dimensional multi-slice gradient echo sequence. Images were acquired at three echo times, with an initial TE of 3.1ms and ΔTE 0.78ms. The flip angle was 5°, and TR was 50ms. The acquisition was obtained in a single breath-hold of 19 seconds. The FOV was 420×300mm^2^, 2.4×2.4mm^2^ acquisition resolution, slice thickness 10mm, slice gap 11.4mm, and five slices were acquired.

### *MRE*

Magnetic resonance elastography was performed using a gravitational transducer^2^, with single frequency mechanical wave generation set at 50Hz. The transducer was positioned in the mid-axillary line adjacent to the liver and held in position using an elastic strap. Image acquisition was performed using a two-dimensional Ristretto MRE sequence, a generalised multi-shot gradient-recalled echo MRE sequence proposed by Guenthner and colleagues^3^. MRE settings were as follows: four wave-phase offsets, motion-encoding gradient frequency 165Hz, Hadamard encoding, Ristretto sequence timing Nw/Nd 5/3. Nine slices were acquired with a FOV of 448 x 448 mm, 4.0x4.0 mm^2^ acquisition resolution and 4.0 mm slice thickness. The flip angle was 20°, TE was 6.91 ms and TR 75 ms. Parallel imaging acceleration factor 3.0 resulted in an acquisition time of four 15 second breath holds.

### *Intravoxel incoherent motion (IVIM) imaging*

IVIM imaging was performed using a free-breathing multi-slice diffusion weighted single-shot echo-planar imaging sequence. Eighteen unique *b-*values were used: 0, 1, 2, 5, 10, 20, 30, 40, 50, 75, 100, 150, 200, 300, 400, 500, 600, and 700 s/mm^2^. The TR was set at 7000 ms, TE 45.5 ms and bandwidth 20.8Hz/pixel. The FOV was 450×295 mm^2^ with a 3.0x3.0 mm^2^ acquisition resolution. 27 slices were imaged with a slice thickness of 6.0 mm and a 1.0 mm slice gap. Scans were accelerated using a parallel imaging factor of 1.3 and partial averaging factor of 0.6. Fat suppression was implemented using spectral attenuated inversion recovery (SPAIR). Three saturation slabs were positioned to suppress signal arising from the anterior abdominal wall. Image acquisition time was 8.1 minutes.

1. Runge JH, Smits LP, Verheij J, et al. MR spectroscopy-derived proton density fat fraction is superior to controlled attenuation parameter for detecting and grading hepatic steatosis. *Radiology*. 2018;286(2):547-556. doi:10.1148/radiol.2017162931

2. Runge JH, Hoelzl SH, Sudakova J, et al. A novel magnetic resonance elastography transducer concept based on a rotational eccentric mass: preliminary experiences with the gravitational transducer. *Phys Med Biol*. 2019;64(4):045007. doi:10.1088/1361-6560/aaf9f8

3. Guenthner C, Sethi S, Troelstra M, Dokumaci AS, Sinkus R, Kozerke S. Ristretto MRE: A generalized multi‐shot GRE‐MRE sequence. *NMR Biomed*. 2019;32(5):e4049. doi:10.1002/nbm.4049

## Supplemental figure legends

***Supplemental Figure 1.*** *FibroScan*® *CAP values versus histological steatosis grade. Median values for grade 1, 2 and 3 were 324, 348 and 336 dB/m resp. There were no significant difference in medians between steatosis grades.*

***Supplemental Figure 2.*** *FibroScan*® *LSM versus histological activity grade. Median stiffness values were: 6.85 kPa for grade 0, 6.93 kPa for grade 1, 10.50 kPa for grade 2 and 11.1 kPa for grade 3. There was no significant difference in medians between activity grades.*

***Supplemental Figure 3.*** *FibroScan*® *LSM versus histological fibrosis grade. Median stiffness values were: 7.05 kPa for grade 0; 7.26 kPa for grade 1; 8.48 kPa for grade 2; 10.40 kPa for grade 3; and 23.00 kPa for grade 4. There were no significant differences in medians between fibrosis grades.*
