# Supplementary figures and images for: Assessment of Imaging Modalities Against Liver Biopsy in Nonalcoholic Fatty Liver Disease: The Amsterdam NAFLD‐NASH Cohort
Source: J Magn Reson Imaging. 2021 May 15;54(6):1937–49. doi: 10.1002/jmri.27703 (PMC9290703; doi:10.1002/jmri.27703)

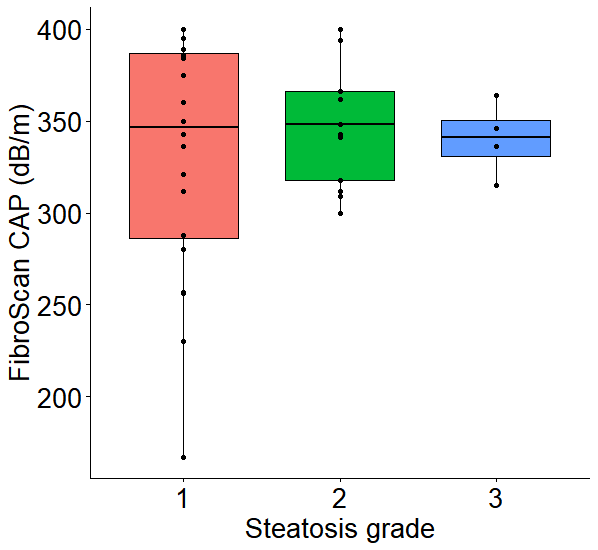

Supplement: Supplementary file 2 — Fig. S1 FibroScan® CAP values versus histological steatosis grade. Median values for grade 1, 2, and 3 were 324, 348, and 336 dB/m resp. There were no significant differences in medians between steatosis grades. [file JMRI-54-1937-s002.tiff]

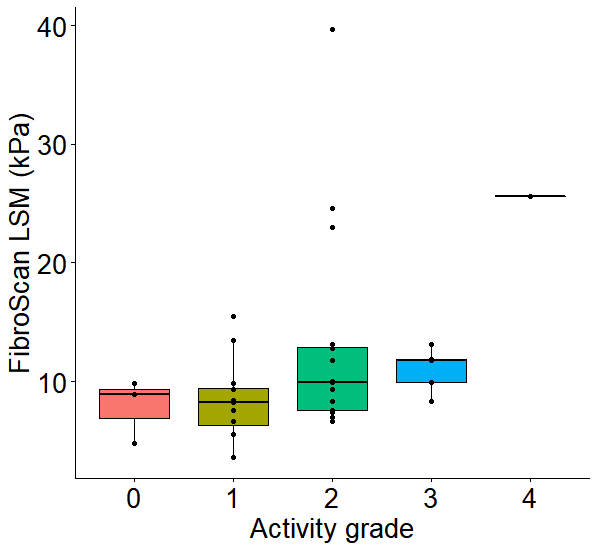

Supplement: Supplementary file 3 — Fig. S2 FibroScan® LSM versus histological activity grade. Median stiffness values were: 6.85 kPa for grade 0, 6.93 kPa for grade 1, 10.50 kPa for grade 2, and 11.1 kPa for grade 3. There was no significant difference in medians between activity grades. [file JMRI-54-1937-s001.tiff]

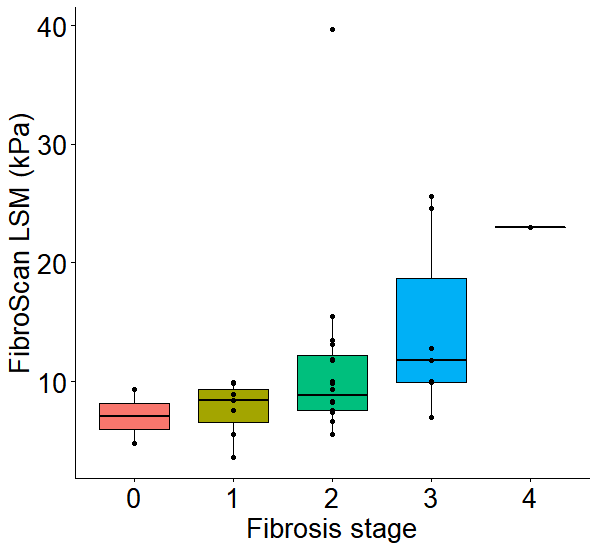

Supplement: Supplementary file 4 — Fig. S3 FibroScan® LSM versus histological fibrosis grade. Median stiffness values were: 7.05 kPa for grade 0; 7.26 kPa for grade 1; 8.48 kPa for grade 2; 10.40 kPa for grade 3; and 23.00 kPa for grade 4. There were no significant differences in medians between fibrosis grades. [file JMRI-54-1937-s004.tiff]
